# Advocacy as a Tool for Advancing Family Planning in the Democratic Republic of the Congo: A Case Study

**DOI:** 10.9745/GHSP-D-23-00021

**Published:** 2023-12-22

**Authors:** Arsene Binanga, Grace Matawa, Jennifer Racher, Protais Musindo, Jane T. Bertrand

**Affiliations:** a Tulane International, Kinshasa, Democratic Republic of the Congo.; b Tulane School of Public Health and Tropical Medicine, New Orleans, LA, USA.

## Abstract

The national strategic plan for family planning (FP) in the Democratic Republic of the Congo (DRC) calls for an increase in modern contraceptive prevalence in 2025. The DRC's size and weak health infrastructure pose significant challenges to programming in all areas of primary health care, including FP. Notwithstanding, in the past decade, a group of advocates organized to undertake a series of activities to improve the policy environment for FP in the DRC. We outline the 4 major accomplishments to date: revitalizing a multisectoral FP stakeholder group at the national and provincial levels, obtaining the repeal of the Law of 1920 in the form of a new Public Health Law passed in 2018, soliciting public statements favorable to FP from political figures including 2 presidents, and securing the disbursement of government funds for the purchase of contraceptives. We reveal the work behind the scenes needed to obtain these advocacy wins. Given that the field of advocacy does not lend itself to rigorous evaluation using randomized trials or quasi-experimental design, it is important to document and analyze how such advocacy wins can be attained.

## Abstract (precis)

This article analyzes the processes used in the Democratic Republic of the Congo to achieve 4 advocacy wins—evidence that systematic efforts at policy advocacy can result in concrete outcomes.

## Teaser

**Key Messages:**

- Advocacy is an effective tool for influencing policy that advances family planning.
- In the Democratic Republic of the Congo, advocacy was an ongoing process, not a one-shot intervention.
- Documentation of the process is important because advocacy doesn't lend itself to impact evaluation using conventional methods.

**Key Implication:**

- Those wishing to increase government commitment to family planning should adopt the advocacy tools that have proven effective in other countries.

## BACKGROUND

In a 2017 article, “The politics of family planning policies and programs in sub-Saharan Africa,” John May described the policy environment at that time in broad strokes, arguing that the region's current high fertility norms were deeply rooted in traditional reproductive health (RH) regimes.^1^ He put forth several reasons for the modest progress in increasing modern contraceptive prevalence in many countries. Foremost were the lukewarm attitudes of African policymakers toward family planning (FP). Some viewed large population size as economically advantageous and cited the low population density as justification for continued growth.^1^ Others questioned the motives of countries that wanted to limit population growth in Africa. Even policymakers who were convinced of the benefits of expanding FP programs may have feared antagonizing their constituencies.

There have been positive signs of change in policy for FP in sub-Saharan Africa. The percentage of African countries that viewed rapid population growth as negative increased from 25% in 1976 to 64% in 2009.^2^ The prospect of the demographic dividend, achievable through declining fertility rate (and other factors), has rekindled interest in FP. In the wake of the 2012 London Summit, multilateral and bilateral donors, as well as private foundations, have infused large amounts of funding into FP in multiple countries across Africa. Advocacy tools such as RAPID and ENGAGE (Eliminating National Gaps—Advancing Global Equity) have improved “demographic literacy” among policymakers across the continent.^3^^,^^4^ Although several countries have achieved a level of contraceptive prevalence well above the mean for sub-Saharan Africa, May concluded that many countries are decades away from “achieving the contraceptive revolution.”^5^

As international donors continued to invest millions of dollars in improving the supply of and demand for quality FP services, there was interest in improving the policy environment for these programs throughout sub-Saharan Africa. A scoping review of interventions assessed to effectively impact contraceptive prevalence use identified advocacy as an example.^6^ May and Rotenberg highlighted the need for coupling a set of policy levers with renewed commitment from sub-Saharan African leaders and increased investments in both FP and population institutions.^7^

In 2009, the Bill & Melinda Gates Foundation funded the Advance Family Planning (AFP) project through the Johns Hopkins University to positively affect the policy environment for FP through evidence-based advocacy in 21 sub-Saharan African countries, as well as India, Indonesia, and the Philippines.^8^ A 9-step SMART advocacy framework guided AFP's work in the Democratic Republic of Congo (DRC), as in other countries (Figure). The objectives and activities for each country were tailored to the local context, with advocacy targets at the national and/or regional level. Over its 10 years of operation, AFP developed a more systematic approach to advocacy through SMART objectives and attempted to connect short-term advocacy strategies and wins with broad, long-term goals.

**FIGURE:**
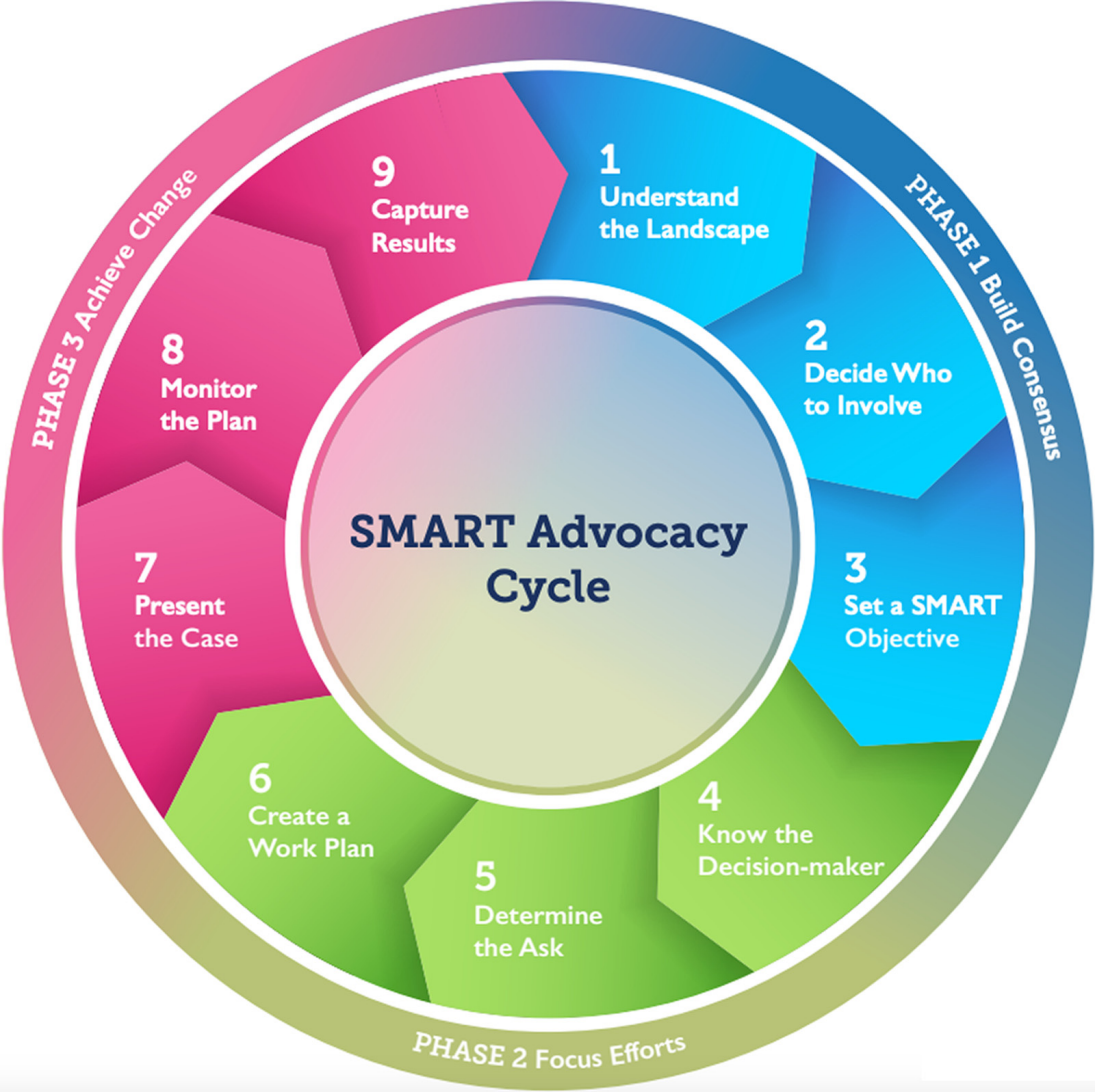
SMART Advocacy Strategy in 9 Steps Source: Advance Family Planning.

Of the 21 sub-Saharan African countries participating in AFP, the DRC was possibly the most challenging. Despite its inestimable mineral wealth and fertile countryside, the country is among the most impoverished in the world, with 62% living on less than US$2.15 a day.^9^ It ranks 176th of 189 on the Human Development Index. Cronyism and financial mismanagement, dating back to the ruinous administration of President Mobutu Sese Seko (1965–1997), are pervasive. Although the government struggles to confront the current major public health issues (e.g., recurring Ebola and COVID-19), the average family struggles for survival.

The DRC is also challenging from a policy perspective. As a vestige of the colonial era (with independence from Belgium in 1960), the Catholic Church still plays a dominant role in the politics of the country and the lives of many of its citizens. Despite the country's mineral wealth, government funding for public health is woefully inadequate at US$18.50 per capita.^10^ As in many other countries, other health issues—malaria, child immunization, HIV/AIDS, and sanitation—are prioritized over FP. As a result, FP in the DRC has historically been almost 100% donor dependent, which has its own problems, including a potential absence of local ownership.^11^ However, donor funding has meant the difference between having viable FP programs in the DRC or not.

Despite these challenges, the AFP efforts in the DRC between 2014 and 2022 have delivered measurable results. Advocacy does not lend itself to meaningful evaluation through randomized control trials or quasi-experimental designs. Rather, it is useful to examine major achievements in advocacy and to analyze how they unfolded as a means of advancing knowledge in this relatively new field.

It is useful to examine major achievements in advocacy and to analyze how they unfolded as a means of advancing knowledge in this relatively new field.

In this article, we document 4 key accomplishments of the AFP project in the DRC and address the critical questions of strategies, audiences, channels, and outcomes. We also describe how the wins were interrelated. Finally, we place these achievements in the context of findings from other countries, especially in francophone sub-Saharan Africa.

## REVITALIZING A MULTISECTOR FAMILY PLANNING STAKEHOLDERS GROUP

Advocacy is needed both at the national level and the provincial level, especially in a vast country with communication, transportation, and logistical challenges. At the close of the 2009 National Conference to Reposition Family Planning in the DRC, there was a recommendation to establish a group of FP stakeholders in the form of a multisectoral permanent technical committee, known locally as the Comité Technique Multisectoral Permanent (CTMP).

Initially, the CTMP group focused on specific tasks, such as organizing a mini-conference to showcase existing FP work, but then focused on advocacy: how to change policies, mobilize resources, and attract new donors. The timing proved fortuitous because the CTMP was beginning to work on persuading the government to publicly pledge its support to FP2020 at the 2013 International Conference on Family Planning (ICFP) meeting in Addis Ababa. As the national CTMP became more active, efforts began in 2015 to expand this mechanism as an advocacy tool for FP at the provincial level. Provincial CTMPs were gradually established to a total of 20 in the 26 provinces as of 2022.

In March 2015, Prime Minister Matata Ponyo Mapon conferred official status on the CTMP through a ministerial decree. The CTMP proved to be an effective mechanism for increasing the visibility of FP on the national health agenda, and it allowed this group of stakeholders to speak with one voice. This official decree proved to be fortuitous because it gave the CTMP leverage to work in a coordinated fashion in achieving the other 3 advocacy wins. The decree also increased donors' confidence that the government was serious about FP, resulting in increased funding and heightened motivation among implementing agencies.

The CTMP is currently donor dependent, as is the vast majority of FP programming in the DRC. However, as a donor-sponsored activity, it is surprisingly inexpensive because it does not have an annual budget and permanent staff. Rather, international nongovernmental organizations (INGOs) that are already funded for FP support CTMP's work at the national and provincial levels. The coordination of the national and provincial levels rests with INGOs experienced in resource mobilization, whereas the Secretariat rests with the Programme National de Santé de la Reproduction (National Program for Reproductive Health, PNSR). The composition of the provincial CTMPs differs by province but includes some combination of the provincial representatives of the PNSR and the Programme National de Santé de l'Adolescent (National Program on Adolescent Health), staff of INGOs, civil society working in the province, and provincial ministerial representatives (Gender/Women/Family/Children, Plan, Budget, Environment, or Finance).

The primary advocacy activity of the provincial CTMPs has been to obtain a line item in the provincial-level budgets to purchase contraceptives. To date, 7 of the 20 provinces have achieved this result, but none of the provinces has released the budgeted funding. Nonetheless, the provincial CTMPs continue to play a valuable role in establishing a presence for FP at the provincial level and maintaining communication among actors working in different organizations.

In late 2018, the CTMP ran into difficulties when new leadership at the PNSR viewed the CTMP as a competitor, not an ally. Two CTMP coordinators resigned from their positions to avoid political backlash. In late 2022, a provisional office based at the United Nations Population Fund (UNFPA) was established to revitalize the CTMP. It proved useful in coordinating the DRC participation in the 2022 ICFP in Thailand, including the public commitment made to the goals of FP2030.

This model of the FP stakeholders' group is not novel. The CTMP draws lessons learned from the Ouagadougou Partnership, which also maintains a network of FP advocates, though across 9 countries, not provinces of a single country. Several countries across sub-Sahara Africa, including Uganda, Kenya, Nigeria, and Tanzania, have similar groups working in tandem with both governments and INGOs on FP advocacy. Although other countries have formal or informal groups advocating for FP, none are set up exactly like the CTMP in the DRC.

In a country where the national agency responsible for FP does not have the human or financial resources to play this coordination role effectively at the national level, the CTMP has been effective in maintaining the engagement of actors across the DRC.

The CTMP has effectively maintained the engagement of advocacy actors at the national level.

## REPEALING THE LAW OF 1920

Among the many obstacles to FP in francophone sub-Saharan Africa is the Law of 1920, a French law that prohibited the distribution of contraception, practice of abortion, and advertisement of contraceptives in an effort to encourage procreation. France applied this law to all of France's colonies in Africa.^12^ In 1923, Belgium enacted a similar law, the Law of 1923,^13^ and extended it to its colony, the Belgian Congo, to increase the population and encourage population growth.^14^ When it gained independence in 1960, the country continued to use the Belgian system of laws, including the Law of 1923.^15^ Because the French law applied to more countries, history has subsumed the Belgian law under the better-known name, Law of 1920.

The first (and ultimately unsuccessful) attempt to repeal the Law of 1920 in the DRC occurred in 1982 (when it was Zaire), when the administrator of the local International Planned Parenthood affiliate, the Association Zaïroise pour le Bien-être Familiale, appeared before the Parliament (Supplement).

After the 2011 Rencontre Internationale Femmes et Santé (Meeting on Women and Health) in Brussels, a local civil society organization of powerful female professionals (Cadre Permanent de Concertation de la Femme Congolaise) began working with UNFPA to develop a proposal for an RH law. Two female deputies, Marie-Ange Lukiana Mufuankolo Dialukupa and Eve Bazaiba Masudi, endorsed the proposal and presented it to the Socio-cultural Commission of the National Assembly; it was presented to the plenary session in June 2014. The launch of the *National Multisectoral Strategic Plan for Family Planning: 2014–2020* brought a renewed sense of urgency to repeal the Law of 1920.

However, the draft law encountered obstacles and was sent to the Supreme Court of Justice to decide whether the DRC needed a specific RH law if a draft of a general health law was in circulation. The CTMP and Cadre Permanent de Concertation de la Femme Congolaise (CAFCO) leadership met with magistrates to explain the need for a law favorable to FP and other RH topics. In 2016, the Constitutional Court released a decision favoring a specific RH/FP law. But 2 years later, the president of Parliament encouraged the FP advocates to drop having a separate law and instead incorporate FP into a proposed new law on public health that the Ministry of Health (MOH) was promoting. The advocates agreed; it diluted some FP provisions but facilitated the acceptance of others.

The Public Health Law, passed in 2018, authorizes FP access for all individuals of reproductive age, stating “All persons of reproductive age – after benefiting from counseling – have the right to voluntarily use a reversible or irreversible contraceptive method” (article 81). “For legally married people, the consent of both spouses on the contraceptive method is required” (implying that the spouses must at least discuss contraceptive use). “In the event of disagreement between the spouses on the contraceptive method to be used, the will of the spouse intending to use the contraceptive takes precedence” (article 82).

The new law had immediate benefits for married women to gain greater autonomy of decision-making in relation to contraception (albeit after discussion with their spouse) and adolescents and youth to have access to contraception, thanks to the phrase “all persons of reproductive age …” However, a major challenge is the difficulty of enforcing the law, especially as it relates to behaviors (conversations) held in private between spouses.

In retrospect, the successful passage of the law was facilitated by three efforts. The team of advocates identified that the president of Parliament had the decision-making power. He directed the team of advocates to others within Parliament who were influential and potential allies. The team produced rationales for the new law, pointing to the contradiction between President Mobutu's speech in 1972 authorizing naissances désirables (“desirable births,” a euphemism for FP) and actual practice. Finally, the team sought the Politique Administratif Juridique, a small group that reviews cases in advance of their being presented for a vote.

The successful passage of a new Public Health Law was facilitated by efforts in identifying key people of influence with decision-making power who could serve as potential allies.

The DRC is among the last of 15 countries in francophone sub-Saharan Africa to repeal the Law of 1920 (Table 1), reflecting the importance that other nations have given to striking this law from the books.

**TABLE 1.** List of Countries and Year in Which They Repealed or Removed the Law of 1920, Prohibiting the Promotion or Distribution of Contraception

Year of Repeal of Law of 1920	Country
1967	Cameroon^16^
1969^a^	Gabon^17^
1972	Mali^18^
1981	Cote D'Ivoire^19^
1981	Senegal^20^
1986	Burkina Faso^21^
1988	Niger^22^
1993	Chad^23^
2000	Guinea^24^
2003	Benin^25^
2006	Togo^26^
2010	The Republic of Congo^27^
2017	Madagascar^28^
2018	DRC

a Although the law was repealed, a new similar law was put in place by the government. This law was reversed in 2000.

## SOLICITING PUBLIC DECLARATIONS ON FAMILY PLANNING

When high-level officials publicly discuss FP in favorable terms, it contributes to a positive policy environment. We describe the main favorable public declarations by DRC political authorities in the last decade.

### DRC's Public Commitment to FP2020 in 2013

Recognizing the importance of public commitments to FP, the London Summit of 2012 provided a platform for governments to pledge. On July 11, 2012, 13 low- and middle-income countries described how their governments planned to support FP in terms of changes in policies, financial support for contraceptive procurement, and barriers to access to contraceptives, such as sociocultural factors and quality of care.^29^^,^^30^ These statements required governments to reconsider the priority given to FP, a topic that had fallen off the global public health radar in many countries. A certain bandwagon phenomenon occurred, whereby countries did not want to be left behind; rather, they actively worked to identify commitments that would allow them to “shine” on the international stage. The international conferences that have taken place since then (ICFP 2013, Addis Ababa; ICFP 2016 Nusa Dua; ICFP 2018, Kigali; and 2019, the Nairobi Summit [ICPD+25]) have provided a space for additional countries to make their commitment. A second London Summit in 2017 gave another opportunity for countries to publicly pledge their commitment to FP.^31^ To date, 46 low- and middle-income countries have made a public commitment at 1 of these international meetings. Some international donors consider a country making a public commitment and having a national strategic plan to be conditions for funding.^29^

In 2013, the CTMP formed a subcommittee on advocacy with its first order of business to persuade the DRC government to publicly commit to FP2020 at the 2013 ICFP in Addis Ababa. The relatively inexperienced advocacy subcommittee benefited from technical assistance from AFP. Together, they convened a workshop on the use of the SMART tool for advocacy, analyzing the steps needed to pursue an advocacy objective. Working with a key ally in the Direction D'Etudes et Planification (DEP), a nonpolitical group of technical advisors in the MOH, they were able to raise awareness of FP to the Prime Minister. CTMP representatives from the UNFPA and the World Bank met with the Prime Minister and his advisers to encourage him to make concrete efforts to prioritize FP in the DRC. Members of the CTMP knowledgeable about FP worked closely with the Prime Minister's advisor, identifying ways in which the pledge would align with his own priorities. Mr. Dieudonné Kwete gave an eloquent speech on the DRC's commitment to FP2020. The declaration of commitment at ICFP 2013 had an immediate impact on advocacy activities in the DRC. Then, in 2015, the Prime Minister issued the ministerial decree giving official status to the CTMP, which, in turn, gave it greater leverage in its subsequent advocacy work.

### Involvement of the Prime Minister and 3 Ministers at ICFP 2016

By the time of the 2016 ICFP in Indonesia, the Prime Minister's office was far more conversant on the issues of population, development, and the demographic dividend. Unable to attend the event in person, the Prime Minister gave his remarks at the closing plenary session via a taped video.

The 2016 ICFP also afforded other advocacy opportunities. The DRC was the only country with 3 ministers attending the conference, and they participated in sessions for high-level officials. Exposing these ministers to other countries' engagement in FP was a form of advocacy because there were multiple voices at high levels advocating for FP. In addition, the DRC organized a satellite session that showcased results achieved and new initiatives (e.g., trying to persuade the mining industry to invest in FP in response to a new law requiring corporate social responsibility).

### President Joseph Kabila's Address in 2018 Citing Need for Strong FP Policies in Search of the Demographic Dividend

In 2018, the government issued a new law for the mining industry (Code Minière) that called for the development of corporate social responsibility in mining companies. The advocacy team saw an opportunity for FP to benefit from this funding.

At the 2019 Mining Company Industrial Fair at Kolwezi in the province of Lualaba, the CTMP advocacy subcommittee received permission to set up a booth at the conference. The advocacy team incorporated President Joseph Kabila's 2018 national address that cited the need for a strong population policy in search of the demographic dividend and his image into their conference materials. The team displayed a large banner at the entrance that referred to the president's discourse on the demographic dividend. At their booth, they repeatedly played an audio tape of this segment of President Kabila's speech, which caught the President's attention as he toured the booths. The national coordinator of the CTMP and the director of the PNSR made a brief pitch to the President, indicating the importance of using corporate social responsibility funding to support FP and including a line item in the provincial budget for contraceptive procurement. President Kabila asked whether they had spoken with the Governor of Lualaba, who was in the President's group and could act on this request.

### President Tshisekedi's 2019 Call for FP as Part of Universal Health Coverage

One of President Félix Tshisekedi platforms was free education. The CTMP advocacy committee wanted his endorsement for the Fourth National Conference to Reposition Family Planning, so they met with the director of the President's Cabinet to explain the strong link between FP and free education (if there are fewer students to educate with limited funding, that money then could be reinvested in education). Through a series of meetings, they identified that the deputy director of the Cabinet responsible for sociopolitical issues worked closely with the President. A U.S. Agency for International Development official—also a member of the CTMP—served as a channel to the U.S. ambassador who had a close relationship with President Tshisekedi to encourage him to speak on the benefits of FP. In a 2019 national address, President Tshisekedi specifically mentioned FP when he advocated for universal health coverage.

Presidents of other countries have also spoken favorably on FP. At ICFP in 2011, former President of Senegal, Abdoulaye Wade, emphasized the importance of FP and education.^32^ Burkina Faso's President, Blaise Compaoré, gave a keynote speech at the 2011 ICFP titled “Population, Development, and Family Planning: The Urgency to Act” that led to the creation of the Ouagadougou Partnership,^33^ which incorporates 9 francophone West African countries that are publicly committed to increasing FP.^34^ However, such public statements on FP by a president of a francophone country remain rare.^1^

## SECURING DISBURSEMENT OF GOVERNMENT FUNDS FOR CONTRACEPTIVE PROCUREMENT

Although positive statements by public officials are advantageous to improving the policy environment for FP, some would argue that “talk is cheap.” In countries worldwide, the purchase of contraception with government funds has become a marker of government commitment to FP. Thus, from the earliest days of AFP, the DRC advocacy team began efforts to persuade the government to establish a line item in the annual MOH budget and, more importantly, to release the funds in that line item for contraceptive procurement.

The advocacy team began efforts to persuade the government to establish a line item in the annual MOH budget and release the funds in that line item for contraceptive procurement.

As early as 2012, the key DEP ally discussed with the CTMP advocacy team the potential opportunity to persuade the government to purchase contraceptives. The government of Prime Minister Matata had developed a program for strengthening health facilities, including the purchase of equipment in 2013. With the launch of the *National Multisectoral Strategic Plan for Family Planning: 2014–2020*, it became clear that FP had a well-organized constituency with a clear vision, likely to attract further donor funding to the country. The DEP included a line of US$300,000 to purchase contraceptives that were delivered in 2015. In 2017, the government subsequently disbursed US$1M for contraceptives. Several years passed with no further disbursement due to the presidential elections in 2018 and the COVID-19 pandemic starting in 2020. Yet, the advocacy team continued to work closely with the Ministry of Finance and MOH to obtain the release of US$2.1M in 2021.

The DRC joins other francophone African countries that established a budget line specifically to purchase contraceptives. Burkina Faso was at the forefront in 2007.^26^ Senegal, Guinea, and Niger established this line item following the ICFP in 2011, the year the Ouagadougou Partnership was established.^34^^–^^36^ Cameroon (in 2017) and Cote D'Ivoire (in 2018) added FP, including contraceptive procurement, to their budget to improve access to contraception and supply chain management.^37^^,^^38^

An important distinction must be made between establishing a budget line item and releasing the funds. Although Niger established the line item in 2012, it has yet to release any funds.^35^ Other countries have decreased the original line item (e.g., Cote d'Ivoire by 10% between 2018 and 2019).^38^ Similarly, other countries have fallen short of their promised level of funding. In Senegal, the government committed to increasing funding for the procurement of commodities by 200%, yet they only increased by 100%.^34^ These gaps in commitments versus implementation result in part from a failure to understand or master the government mechanisms for disbursement of government funds.^39^ They may also reflect inadequate political will.

Table 2 illustrates the 9-step advocacy process (Figure) and how it was applied to obtaining the release of government funds for contraceptive procurement.

**TABLE 2.** The 9 Steps in the Advocacy Process Applied to Securing the Government Disbursement of Funds for Contraceptive Procurement

Phase, Step, and Strategy	Details of Each Step in the Process
Build consensus	
1. Understand the landscape	A landscape analysis identified advocacy opportunities: The government had previously released funds for contraceptive procurement in 2013 and 2017. A line item was created in the national budget in 2015, but no funds had been released since 2017.
2. Decide who to involve	Minister of Health, Minister of Finance, Director of DEP, UNFPA, Tulane International, Options (United Kingdom)
3. Set a SMART objective	Release of funds by the DRC government for the purchase of contraceptives in 2021
Focus efforts	
4. Know the decision-makers	Minister of Health, Minister of Finance, and Director of DEP
5. Determine the ask	The request for US$2.1M of the US$4.4M in the line item for contraceptive procurement (tranche for first trimester) in 2021
6. Create a work plan	Key meetings were held with: PNSR to prepare statement of contraceptive needs DEP to monitor the dossier with the Ministers of Health and Finance UNFPA to develop the plan for purchasing the contraceptives DEP and UNFPA to monitor and confirm the disbursement of funds for contraceptive procurement
Achieve change	
7. Present the case	The advocacy brief contained: A statement of contraceptive needs prepared by PNSR The costs for this quantity of contraceptives, prepared by UNFPA A letter of commitment signed by the Minister of Health Various official communications (e.g., letters and instruction notes)
8. Monitor the plan	The advocacy team established indicators of progress, consistent with the SMART framework. Each quarter, the team assessed program against these indicators (delayed, in progress, completed, or abandoned).
9. Capture results	When the government released US$2.1M to UNFPA for contraceptive procurement, this event was announced: By the Minister of Health in his speech at the ICFP 2022 in Pattaya, Thailand As a “win” in the AFP database

Abbreviations: AFP, Advance Family Planning; DEP, Direction d'Etudes et Planification (Department of Research and Planning); DRC, Democratic Republic of the Congo; ICFP, International Conference on Family Planning; PNSR, Programme National de Santé de la Reproduction (National Program for Reproductive Health); SMART, specific, measurable, attainable, relevant, and time-bound; UNFPA, United Nations Population Fund.

## DISCUSSION

Although we present these 4 advocacy wins as discrete events, they formed part of a continuous process whereby local advocates remained alert to opportunities in both an intentional and opportunistic way.

There is little literature available on the value of an FP stakeholders' group like the CTMP at the national level for francophone sub-Saharan African countries, in large part because 9 of the countries in this region form part of the Ouagadougou partnership, which plays a similar role in promoting a favorable environment for FP. CTMP's value in the DRC relates to its role in establishing an identified network of FP champions and the motivation derived from a collective *esprit de corps*. Despite this very encouraging model, CTMP suffered a considerable setback from late 2018 to early 2022 but appears to be reorganizing at the national level and continues to play an important role at the provincial level.

Several important lessons can be drawn from the DRC experience. First, regarding the repeal of the Law of 1920, the president of the National Assembly recommended that advocates incorporate their issues on FP into a more general public health law. Although they sacrificed several minor points in doing so, the advocacy team succeeded in achieving their main goal of making “contraception accessible to all individuals of reproductive age.” If they had used the wording “including unmarried adolescents,” they would surely have met with far greater resistance. Their acceptance of this wording change allowed them to achieve their objective while avoiding a cantankerous debate over the morals of young women. There was great benefit in focusing on the essence of the outcome, even if the outcome was not exactly as desired. However, in winning this major concession, they had to concede on wording related to permanent sterilization, “In the case of irreversible contraception, the client must provide written consent, after consulting with 3 doctors and the psychiatrist.”

A second important lesson for heightening the visibility of FP and convincing decision-makers of its importance was to link FP to other government plans and health policy documents such that it appeared in support of other government initiatives and priorities. Table 3 lists the official documents issued between 2013 and 2020 in which FP is cited.

**TABLE 3.** Official Documents in the Democratic Republic of the Congo That Cite Family Planning

Year	Document
2013	Projet D'équipement des Structures Sanitaires (Health Facility Equipment Project)
2014	Education Strategy, Ministry of Education
2014	Priority Action Program
2015	Prime Minister Decree N°15/003 of March 6, 2015, relating to the creation and operation of the Comité Technique Multisectoral Permanent
2015	Programme National de Santé de l'Adolescent (National Program for Adolescent Health) Strategic Plan 2016–2020
2015	Plan National de Développement Sanitaire 2016–2020 (National Health Development Plan)
2015	Law on the Implementation of Women's Rights and Gender Parity, Article 14
2016	Global Financing Facility Investment Framework
2016	Amendment to the Family Code
2018–2020	Medium-Term Budgetary Framework

Third, the multisectoral nature of this work—explicitly stated both in the name of the Strategic Plan and the stakeholders' group—broadens the appeal of its reach to include other constituencies. Family planning advocates collaborated closely with the Minister of Gender in obtaining passage of the Public Health Law of 2018. Similarly, those working to obtain the release of government funds for contraceptive procurement have worked through the Ministry of Finance.

In discussing the effectiveness of public commitment to advancing FP, it is important not to overlook the issue of ownership. Appleford and Emmert analyzed the extent to which national FP programs benefited from their engagement with FP2020.^39^ They concluded that not all countries were able to convert FP2020 commitment into national ownership.

*In many FP2020 contexts, there is less need for a technical intervention and greater need for engaging politically on sensitive issues that constrain women's and adolescent empowerment and rights and access to FP.*

In a field where the impact of interventions cannot be evaluated using randomized experiments or quasi-experimental design, it is nonetheless important to compile the evidence on the results obtained from systematic efforts at policy advocacy in FP. Given the challenges inherent in development work in the DRC, the results achieved to date merit documentation as a model for other countries in the region.

Although advocacy efforts in the DRC have achieved these wins, much remains to be done. Priority objectives for the future include the following.

- Obtain from the DRC government the automatic replenishment and disbursement of the budget line for the purchase of contraceptives to be directed to the PNSR and the D6 (Direction de l'Enseignement des Sciences de Santé [the Directorate for Health Sciences]) for use in nursing schools. By doing so, the DRC government will substantially reduce the contraceptive gap and will encourage donors to commit to purchasing additional contraceptives.
- Increase private sector investment in FP as part of corporate social responsibility (e.g., with the mining sector, whose corporate social responsibility code was amended in 2018, and the telecommunication companies for the broadcast of FP messages).
- Incorporate FP as a priority in government policy documents related to universal health coverage.
- Increase both domestic and international donor funding for FP in the DRC.
- Invest in media advocacy as a strategy to increase engagement of officials, donors, and policymakers in advancing the agenda of FP in the DRC.

## Supplementary Material

23-00021-Bertrand-Supplement.pdf

## Notes

**Peer Reviewed**

**First published online:** xxxx.
