# Supplementary material for: Advocacy as a Tool for Advancing Family Planning in the Democratic Republic of the Congo: A Case Study
Source: Glob Health Sci Pract. 2023 Dec 22;11(6):e2300021. doi: 10.9745/GHSP-D-23-00021 (PMC10749657; doi:10.9745/GHSP-D-23-00021)
Supplement: 23-00021-Bertrand-Supplement.pdf [file 23-00021-Bertrand-Supplement.pdf]

Supplement to: Binanga A, Matawa G, Racher J, Musindo P, Bertrand JT. Advocacy as a tool for advancing family planning in the Democratic Republic of the Congo: a case study. *Glob Health Sci Pract*. 2023;11(6):e2300021. https://doi.org/10.9745/GHSP-D-23-00021

Supplement. Dr. Miatudila and the Law of 1920

In his 1982 address to Parliament, Dr. Miatudila intended to argue that the Law of 1920 had become obsolete: if a law is repeatedly violated over time with implicit approval of the authorities, it becomes c*aduque* (has lapsed). He delicately began to address the topic, but before he could complete his argument, one of the Parliamentarians began to lash into the colleague who had secured the invitation for him to present his case. "Who invited this lout to speak before such as august body as the Parliament? He should be carried off to M*akala* (the Kinshasa Central Prison), because what he is saying is blatantly against the law."

To this, Miatudila answered in a soft voice, "Excuse me, I am only a humble doctor. Where is the law written down?"

The Parliamentarian shot back, "in this book of laws," which he hoisted over his head for all to see.

Miatudila had anticipated this exchange and had brought his own copy of the *Code Penal du Congo Belge et du Rwanda-Urundi.* "I too have a copy of your book of laws. So let me read to you from it." He opened to a passage and began to read to the assembled members of Parliament, "*il est interdit d'habiter dans le quartier des blancs.*" (It is prohibited [for Congolese] to live in the zone inhabited by the whites.) "*Les prisonniers congolais sont tenus de laver les vêtements sales des prisonniers blancs."* (The Congolese prisoners are required to wash the dirty clothes of the white prisoners.") This book of laws clearly dated to the days of colonial rule, which were abhorrent to the now independent citizens of Zaire. In a flash, the atmosphere changed; the Parliamentarians began to applaud. With an approving gesture, they added: "You can let him leave."

Although Miatudila avoided being whisked off to Makala, he was unsuccessful in having the Law of 1920 stricken from the books. It would take another 36 years before Parliament passed the 2018 Public Health Law that legalized access to contraception for all people of reproductive age.

Source: Bertrand JT. *Fifty Years of Family Planning in the Democratic Republic of the Congo: The Dogged Pursuit of Progress*. Routledge; 2023 (forthcoming).
